# Clinical characteristics and risk factor analysis of overlapping syndromes of COPD-OSA with metabolic syndrome in middle-to-high altitude areas

**DOI:** 10.3389/fmed.2025.1680584

**Published:** 2025-10-08

**Authors:** Yisha Qin, Aniu Aju, Yilin Wan, Mengchen Song, Xingxing Hao, Jiwu Li, Xuefeng Shi

**Affiliations:** 1 Department of Clinical Medicine, Qinghai University, Xining, China; 2 Respiratory and Critical Care Medicine Department, Luoyang First People's Hospital, Luoyang, China; 3 Respiratory and Critical Care Medicine Department, Qinghai Provincial People's Hospital, Xining, China

**Keywords:** middle-high altitude, overlapping syndromes, metabolic syndrome, risk factors, clinical characteristics

## Abstract

**Objective:**

To investigate the clinical characteristics and risk factors of overlap syndrome (OVS) patients with metabolic syndrome (Mets) in middle-high altitude areas.

**Methods:**

A retrospective analysis was performed on adult (≥40 years) OVS patients and healthy controls from Qinghai Provincial People’s Hospital (January 2017–January 2024), including general and laboratory data.

**Results:**

1. OVS patients had a higher rate of Diabetes, Hypertension, and Pulmonary Hypertension than healthy individuals; 2. OVS patients had significantly higher levels of inflammatory markers, hematologic, and lipid than Healthy individuals; 3. The proportion of OVS patients who also had Mets was 55.24%; 4. Compared to OVS patients without Mets, OVS patients with Mets had significantly higher levels of neutrophils, hemoglobin, red blood cell distribution width, C-reactive protein, NHR, and NLR, as well as a higher percentage of time with pulse oxygen saturation (SpO_2_) less than 80%, while the average and lowest SpO_2_ were significantly lower; 5. Hypoxic index, average SpO_2_, baseline SpO_2_, SpO_2_ less than 90%, and SpO_2_ less than 80% may be risk factors for the co-occurrence of OVS and Mets; 6. The rate of Mets among OVS patients who lived at an altitude of ≥2,500 meters was 63.79%, higher than OVS patients who lived at an altitude of <2,500 meters (44.68%).

**Conclusion:**

Over half of middle-high altitude OVS patients have Mets, with higher rates at higher altitudes. Hypoxia may drive OVS-Mets comorbidity, while inflammation appears less significant.

## 1 Introduction

Chronic Obstructive Pulmonary Disease (COPD) is a chronic respiratory disorder defined by irreversible airflow limitation. Globally, it ranks as the third leading cause of death, underscoring its substantial public health burden. Obstructive Sleep Apnea/Hypoventilation Syndrome (OSA), by contrast, is primarily characterized by recurrent breathing pauses during sleep—episodes that trigger intermittent hypoxia (IH), alongside nocturnal hypoxemia and fluctuations in intrathoracic pressure. Clinically, OSA is closely linked to an increased risk of comorbidities such as diabetes mellitus and hypertension; epidemiologically, it is highly prevalent, with an estimated 936 million adults aged 30–69 worldwide affected by mild-to-severe obstructive sleep apnea. The concept of Overlap Syndrome (OVS) was first proposed in 1985, referring to the coexistence of COPD and OSA. Critically, these two conditions do not merely present as a “simple sum” of their individual manifestations-instead, they exert a mutually exacerbating effect that accelerates disease progression and deterioration. Consequently, OVS is associated with a far worse prognosis than either COPD or OSA alone.

Research has shown that Mets may be one of the most clinically relevant factors for COPD (1). In patients with chronic obstructive pulmonary disease (COPD), the prevalence of Mets ranges from 21 to 58%, depending on disease severity, geographical location, and the assessment indicators and methods used (2). Intermittent hypoxia is a fundamental feature of OSA. In patients with OSA, repeated episodes of hypoxemia and hypercapnia during sleep can damage multiple systems, including the endocrine-metabolic, nervous, digestive, and circulatory systems, leading to abnormalities in metabolic, cognitive, immune, and other functions. Additionally, the hypoxic environment specific to high altitudes significantly reduces the blood SpO_2_ of healthy individuals compared to those at low altitudes. This altitude-related hypoxia may further increase the incidence of OVS by impairing systems such as the endocrine-metabolic system. Therefore, this study aims to analyze the clinical characteristics of patients with OVS in middle-to-high altitude areas and identify the risk factors for the coexistence of Mets in these patients.

## 2 Objectives and methods

### 2.1 Study population

This study retrospectively included adult patients (≥40 years old) with COPD who were treated at the Qinghai provincial people’s hospital from January 1, 2017-a Grade A Tertiary Hospital located in Xining, Qinghai, China, with a comprehensive sleep medicine department and a large-scale general outpatient clinic.

Patients were consecutively enrolled in the study. A total of 467 COPD patients and 126 healthy individuals were initially screened from the hospital’s electronic medical record system (inpatient and outpatient departments). After applying the exclusion criteria-including patients who refused to undergo Polysomnography (PSG), echocardiography, pulmonary function tests, or had significant missing covariates-the final cohort consisted of 105 eligible OVS participants and 126 healthy individuals. We have also supplemented a post-hoc power analysis based on the existing sample size, and the results show that the statistical power of this study in the analysis of the risk factor of OVS with Mets (SpO_2_ at baseline Proportion of time when SpO_2_ ≤ 90% during night sleep) reaches 0.96, meeting the basic requirements of clinical research. The PSG scores were analyzed according to the standardized criteria recommended by the American Academy of Sleep Medicine (3).

The healthy control group was recruited following strict inclusion and exclusion criteria: (1) no self-reported history of sleep disturbances (including snoring, daytime somnolence, or previously diagnosed sleep disorders such as OSA); (2) no history of chronic diseases closely associated with OSA pathogenesis (e.g., obesity with body mass index ≥30 kg/m^2^, or upper airway structural abnormalities such as tonsillar hypertrophy); (3) normal findings in routine physical examinations (including vital signs, cardiopulmonary auscultation, and head–neck region inspection for obvious upper airway narrowing). Notably, objective diagnostic tests for OSA—including overnight polysomnography (PSG, the gold standard for OSA diagnosis) were not performed for participants in the healthy control group. This decision was made due to two key constraints: first, the large sample size of the healthy cohort (*n* = 126), which would have resulted in excessive resource consumption (e.g., equipment, personnel, and financial costs) for universal objective testing; second, ethical and practical considerations regarding the burden of overnight monitoring on asymptomatic healthy participants, which could have reduced recruitment efficiency and follow-up compliance.

**Figure 1:**
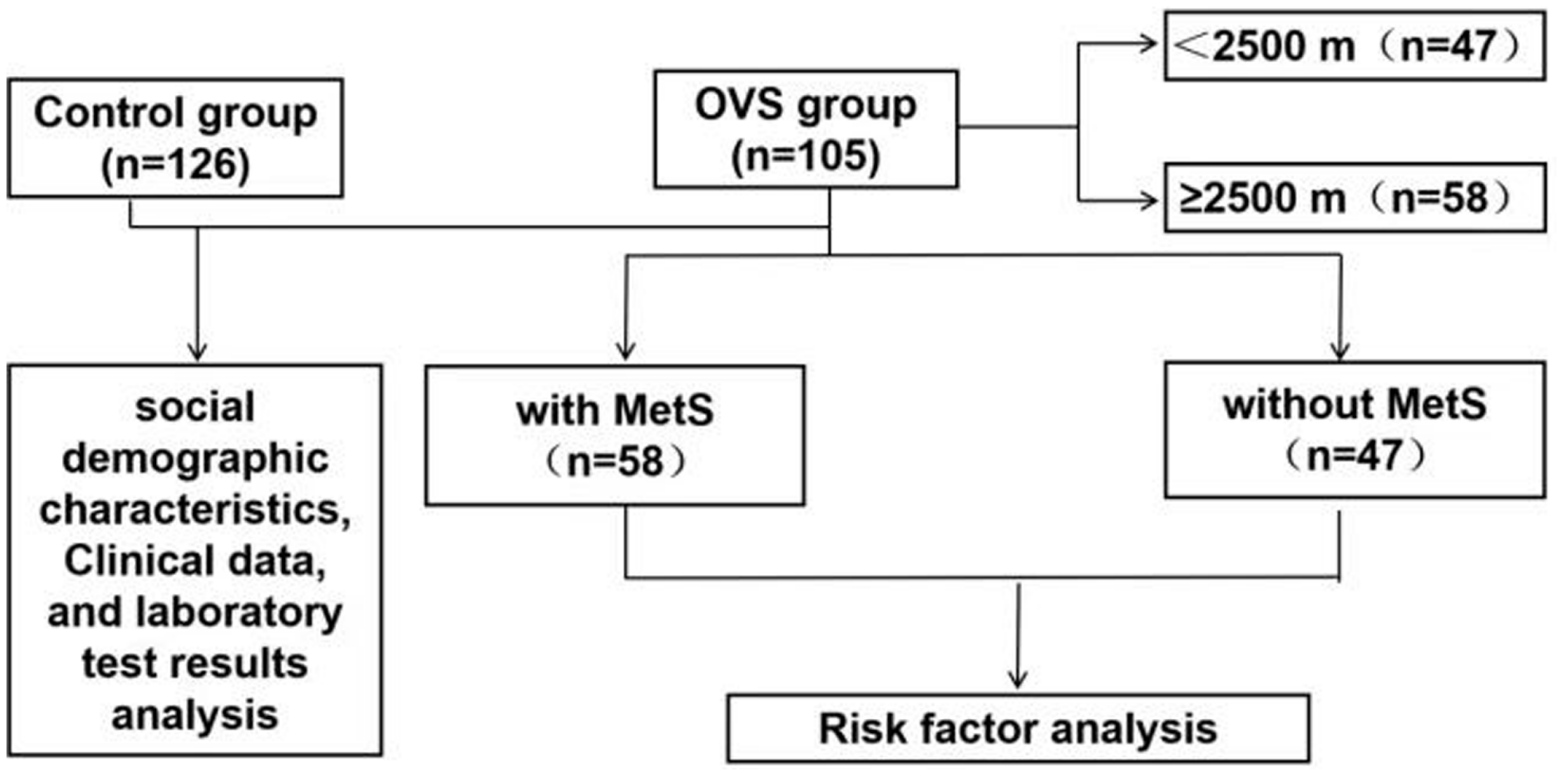
Flow chart.

COPD was diagnosed in accordance with the GOLD 2023 guidelines, which include spirometric criteria (post-bronchodilator FEV1/FVC < 0.7) as well as relevant clinical manifestations. OSA was defined by an apnea-hypopnea index (AHI) of 5 events or more per hour based on AASM 2012 criteria. Therefore, OVS was defined as the coexistence of COPD and OSA, with specific reference to the diagnostic thresholds for both conditions (AHI ≥ 5 events/h for OSA and meeting COPD spirometric criteria as above). The diagnosis of Mets in our study was based on the National Cholesterol Education Program Adult Treatment Panel III (NCEP ATP III) guidelines (2005). This study was approved by the Research Ethics Committee of the Qinghai provincial people’s hospital (No. 2024–024-02). The flowchart was shown in Figure 1.

### 2.2 Data collection

Collecting all social demographic characteristics of the patients, including age, gender, and smoking status, etc. Clinical data, including patient history, such as diagnosis of hypertension, metabolic diseases, pulmonary arterial hypertension; laboratory test results, including complete blood count, lipid profile, etc.; and collect anthropometric indices. After patients use bronchodilators, measure lung function using a lung function instrument (Jaeger; Germany). Parameters include FEV1, FVC, FEV1/FVC, and the predicted percentage of FEV1 (FEV1%pre). Record polysomnography parameters, including apnea-hypopnea index (AHI), sleep stages, oxygen desaturation index, SpO_2_, mean SpO_2_, lowest SpO_2_, etc. According to the Qinghai International Consensus Scoring System (4), divide patients with OVS into those at altitudes of 1500-2499 meters and those at altitudes≥2,500 meters.

### 2.3 Statistical analysis

The analysis of the study results were conducted by SPSS 19.0 software. Count data were compared between groups using the chi-square test. Continuous data that passed the normality test were expressed as mean ± standard deviation ( $\bar{x}$ ± s), and independent sample t-tests were used to compare two groups. Non-normally distributed continuous data were expressed as M (Q1, Q3). For non-normally distributed or heteroscedastic data, the Mann–Whitney U test was used. The comparison of rates between two groups was conducted using the chi-square test for a 2×2 table. Body mass index, oxygen deficit index, lowest SpO_2_, and mean SpO_2_ were included in a binary logistic regression model to assess the risk factors for patients with OVS and Mets. Multicollinearity among the independent variables also was evaluated in the logistic regression model. *p* < 0.05 is considered statistically significant.

## 3 Result

### 3.1 Sociodemographic characteristics and disease history comparison

As shown in Table 1, there were no statistically significant differences in gender or age between the control group and patients with OVS. However, a difference was observed in smoking history. Patients with OVS had a higher body mass index (BMI) and a greater likelihood of comorbidities—including diabetes, hypertension, and pulmonary arterial hypertension-and these differences were statistically significant (*p* < 0.05).

**Table 1:** Sociodemographic characteristics and disease history comparison.

Variables	Control group (*n* = 126)	OVS group (*n* = 105)	*p* value
Gender			0.290
Males	90	82	
Females	36	23	
Age (years)	56.44 ± 5.52	58.50 ± 10.60	0.074
Smoking status			**0.040**
Current smoker	24 (19.0)	31 (29.5)	
Former smoker	23 (18.3)	21 (20.0)	
Never smoked	79 (62.7)	53 (50.5)	
BMI (kg/m^2^)	24.10 ± 2.76	26.95 ± 4.63	**0.000**
Disease history			
Diabetes	2 (1.6)	13 (15.8)	**0.001**
Hypertension	4 (3.17)	54 (51.4)	**0.000**
Pulmonary hypertension	0 (0)	14 (13.3)	**0.000**

Bold values indicate statistically significant differences between groups.

### 3.2 Distribution of laboratory examination indexes of OVS patients

As shown in Table 2, compared with the control group, the levels of neutrophils, neutrophil percentage, monocytes, monocyte percentage, red blood cells, hemoglobin, red cell distribution width, platelets, platelet distribution width, total cholesterol, fasting blood glucose, creatinine, uric acid, monocyte/high-density lipoprotein (MHR), neutrophil/high-density lipoprotein (NHR), and neutrophil/lymphocyte ratio (NLR) were significantly increased in patients with OVS, while the number of lymphocytes decreased, with statistical significance, suggesting that OVS may be closely related to hyperglycemia, hyperuricemia, polycythemia et al. In addition, elevated levels of inflammatory markers such as neutrophils, neutrophil percentage, MHR, NHR, and NLR in OVS patients indicated an increased inflammatory level in OVS patients, which may participate in the progression of the disease in patients with OVS.

**Table 2:** Distribution of laboratory examination indexes of OVS patients.

Variables	Control group (*n* = 126)	OVS group (*n* = 105)	*p*
White blood cell count (×10^9^/L)	5.78 (4.89, 6.98)	5.94 (4.87, 7.32)	0.334
Neutrophil count (×10^9^/L)	3.34 (2.77, 4.42)	3.56 (2.87, 4.62)	**0.021**
Neutrophil (%)	59.8 (53.43, 64.33)	61.70 (54.90, 66.70)	**0.000**
Monocyte count (×10^9^/L)	0.40 (0.32, 0.48)	0.45 (0.34, 0.55)	**0.000**
Monocyte (%)	6.70 (5.90, 7.33)	7.20 (6.20, 8.50)	**0.000**
Red blood cell count (×10^12^/L)	5.45 (5.05, 5.75)	5.59 (5.13, 6.21)	**0.000**
Hemoglobin (g/L)	170 (167, 211)	174 (161, 194)	**0.000**
Red cell distribution width (fL)	44.20 (42.65, 46.40)	46.15 (43.20, 49.70)	**0.000**
Platelets (×10^9^)	195.50 (159.50, 224.25)	171 (96, 197)	**0.000**
platelet distribution width (%)	13.50 (11.87, 16.00)	13.30 (11.28, 16.00)	**0.026**
Total cholesterol (mmol/L)	4.58 (3.82, 5.33)	5.13 (4.48, 5.67)	**0.000**
Triglyceride (mmol/L)	1.81 (1.09, 2.68)	1.71 (1.08, 2.50)	0.143
HDL-C (mmol/L)	1.19 (1.03, 1.43)	1.07 (0.92, 1.29)	**0.000**
LDL-C (mmol/L)	2.99 (2.47, 3.50)	2.65 (2.13, 3.32)	**0.000**
Fasting blood glucose (mmol/L)	4.98 (4.65, 5.31)	5.08 (4.62, 5.57)	**0.003**
MHR	0.34 (0.24, 0.45)	0.52 (0.43, 0.71)	**0.000**
NHR	2.80 (2.01, 4.04)	4.10 (3.04, 5.41)	**0.000**
NLR	1.91 (1.49, 2.50)	2.56 (1.96, 3.35)	**0.000**

MHR, Monocyte/High-density lipoprotein; NHR, Neutrophil/High-density lipoprotein; NLR, Neutrophil/Lymphocyte ratio. Bold values indicate statistically significant differences between groups.

### 3.3 Sociodemographic characteristics and distribution of laboratory examination indexes of OVS patients with or without Mets

The OVS patients were divided into two groups based on whether they were accompanied by Mets: OVS patients with Mets group and OVS patients without Mets group. Results showed that the proportion of OVS patients with Mets was 55.24%. There were no statistically significant differences in gender, age, ethnicity, smoking history, body mass index, neck circumference, and waist circumference between the two groups (*p* > 0.05). In addition, the levels of neutrophils, hemoglobin, red cell distribution width (RDW), C-reactive protein (CRP), NHR, and NLR, as well as proportion of time when SpO_2_ ≤ 80% during night sleep in the OVS patients with Mets were significantly higher than in the OVS patients without Mets, while the mean SpO_2_ and the lowest SpO_2_ were significantly lower in OVS patients with Mets than in the OVS patients without Mets (*p* < 0.05). Although proportion of time when SpO_2_ ≤ 90% and SpO_2_ ≤ 85% during night sleep was higher in the OVS patients with Mets than in the OVS patients without Mets, the differences were not statistically significant (Tables 3, 4).

**Table 3:** Sociodemographic characteristics of OVS patients with or without Mets.

Variables	OVS without Mets (*n* = 47)	OVS with Mets (*n* = 58)	*p*
Gender			0.481
Males	47	35	
Females	11	12	
Age (years)	58.23 ± 11.05	58.71 ± 10.31	0.821
Smoking status			0.149
Current smoker	14	17	
Former smoker	6	16	
Never smoked	27	25	
BMI (kg/m^2^)	26.94 ± 4.72	26.97 ± 4.60	0.973
Neck circumference (cm)	40.72 ± 2.55	40.62 ± 2.15	0.823
Abdominal circumference (cm)	112.57 ± 11.34	110.86 ± 10.34	0.421
Altitude(m)	2593.74 ± 449.51	2608.83 ± 550.11	0.877
AHI	21.15 ± 14.60	21.21 ± 13.96	0.984

**Table 4:** Distribution of laboratory examination indexes of OVS patients with or without Mets.

Variables	OVS without Mets (*n* = 47)	OVS with Mets (*n* = 58)	*p*
White blood cell count (×10^9^/L)	6.02 (4.58,7.34)	6.10 (5.06, 7.76)	0.208
Neutrophil count (×10^9^/L)	3.50 (2.62, 4.65)	3.92 (3.27, 5.19)	**0.041**
Red blood cell count (×10^12^/L)	5.82 (5.21, 6.80)	6.51 (5.35, 7.28)	0.117
Hemoglobin (g/L)	180 (165, 212)	208 (171, 224)	**0.024**
Red cell distribution width (fL)	48.00 (44.00, 53.90)	51.45 (47.08, 60.73)	**0.028**
Platelets (×10^9^)	160.00 (120.00, 206.00)	139.00 (97.00, 199.00)	0.419
C-reactive protein	0.26 (0.12, 0.48)	0.47 (0.21, 1.12)	**0.005**
MHR	0.51 (0.32, 0.65)	0.57 (0.45, 0.72)	0.069
NHR	3.89 (2.71, 5.11)	4.29 (3.53, 6.02)	**0.024**
NLR	2.47 (1.73, 3.06)	2.63 (2.09, 3.55)	**0.045**
ODI4/h	28.40 (14.80, 50.80)	19.55 (11.50, 41.03)	0.189
SpO_2_ at baseline	86.00 (83.00, 89.00)	83.50 (80.00, 87.00)	0.028
Average SpO_2_	84.00 (81.00, 87.00)	83.00 (80.00, 86.00)	**0.218**
Minimum SpO_2_	68.00 (60.00, 77.00)	65.00 (56.00, 71.25)	**0.035**
Proportion of time when SpO_2_ ≤ 90% during night sleep	85.50 (58.90, 95.90)	91.00 (77.03, 97.50)	0.070
Proportion of time when SpO_2_ ≤ 85% during night sleep	42.10 (13.10, 77.40)	59.60 (27.98, 86.83)	0.077
Proportion of time when SpO_2_ ≤ 80% during night sleep	6.20 (0.13, 31.00)	12.25 (3.35, 47.20)	**0.028**

Bold values indicate statistically significant differences between groups. ODI4/h, Oxygen Desaturation Index is 4 events per hour.

### 3.4 Analysis of risk factors in OVS patients with Mets

OVS patients were divided into those with and without Mets based on the presence of Mets. Binary logistic regression models were employed to assess risk factors for OVS with Mets, incorporating neutrophil count, red blood cell distribution width, NLR, NHR, oxygen desaturation index, minimum SpO_2_, mean SpO_2_, baseline SpO_2_, proportion of time when SpO_2_ ≤ 90 and 80% during night sleep. Multicollinearity analysis indicated that the variance inflation factor (VIF) and tolerance values for all variables fall within acceptable ranges, with all VIFs<10 and tolerances>0.1. In addition, results showed that the oxygen desaturation index, mean SpO_2_, baseline SpO_2_, proportion of time when SpO_2_ ≤ 90%, and SpO_2_ ≤ 80% may serve as risk factors for overlapping syndrome with Mets (Table 5).

**Table 5:** Risk factors analysis of comorbid Mets in patients with overlapping syndrome groups.

Variables	*β*	S.E	Wals	*p*	OR(95%CI)
Average SpO_2_	−0.435	0.193	5.089	0.021	0.625(0.419, 0.933)
SpO_2_ at baseline	0.381	0.151	6.330	0.013	1.485(1.085, 2.030)
Proportion of time when SpO_2_ ≤ 90% during night sleep	−0.050	0.019	6.575	0.008	0.945(0.906, 0.985)
Proportion of time when SpO_2_ ≤ 80% during night sleep	−0.048	0.022	4.861	0.025	0.951(0.910, 0.994)

### 3.5 Incidence of Mets in patients with overlapping syndrome at different altitudes

To clarify whether there are differences in the incidence of Mets in patients with overlapping syndrome at different altitudes, this study took 2,500 meters as the boundary and explored the incidence of Mets in patients with overlapping syndrome at different altitudes. The results showed (Table 6) that the incidence of Mets in patients with overlapping syndrome above 2,500 meters was 63.79%, higher than that in the lower altitude group below 2,500 meters (44.68%, *p* = 0.05).

**Table 6:** The incidence of Mets in patients with overlapping syndrome at different altitudes.

	Altitude≤2,500 m	Altitude>2,500 m
With Mets (n)	21	37
Without Mets (n)	26	21
Total (n)	47	58
Incidence rate (%)	44.68%	63.79%

## 4 Discussion

Sleep exerts a significant influence on respiration and gas exchange-effects that can have adverse impacts on patients with COPD (5). Notably, certain respiratory disorders (including OSA) exhibit specific associations with sleep. Given the high prevalence of both COPD and OSA, OVS-defined as the coexistence of these two conditions—is relatively common in clinical practice.

Extensive evidence confirms that COPD and OSA interact significantly, with these interactions shaping key clinical outcomes such as disease epidemiology, comorbidity profiles, and patient survival rates. Epidemiologically, OVS affects 1–3.6% of the general population; however, this proportion rises markedly when assessed within clinical cohorts of patients with OSA or COPD alone (6). A critical mechanistic link between the two diseases lies in their shared ability to induce local and systemic inflammatory responses. These inflammatory processes may act synergistically to drive the development of comorbidities such as Mets (7).

COPD and OSA are associated with heightened cardiovascular morbidity and mortality (8, 9), primarily due to a combination of factors: intermittent hypoxia, systemic inflammation, elevated oxidative stress, metabolic abnormalities, and activation of the sympathetic nervous system (10–12).

Numerous studies have reported that high levels of red blood cell distribution width (RDW) are similar to inflammatory factors such as C-reactive protein (CRP), interleukin, and tumor necrosis factor-*α*, and can reflect the body’s inflammatory levels, making it a novel inflammatory marker (13). In addition, the MHR, NHR, and NLR are also considered inflammatory markers (14, 15). Additionally, studies have demonstrated the predictive value of RDW in relation to pulmonary embolism, as well as its association with the severity and prognosis of this condition (16). The present study revealed significantly higher levels of neutrophils, neutrophil percentage, monocytes, monocyte percentage, RDW, MHR, NHR, and NLR in OVS patients compared to healthy individuals. These findings indicate an enhanced inflammatory response in OVS patients, which may contribute to the development of cardiovascular complications (17). Consistent with this inflammatory profile, the study also found that the incidence of hypertension and pulmonary arterial hypertension was significantly higher in OVS patients than in healthy controls.

A growing body of research highlights a bidirectional correlation between COPD and Mets, with the latter exacerbating the progression and prognosis of the former. Additionally, COPD may be a significant risk factor for the development and persistence of Mets. This correlation is primarily reflected in the presence of hypoxia and chronic inflammation in patients with COPD-two key pathological features that may contribute to the development of Mets. Furthermore, diabetes and hypertension are two major complications of OSA. Studies have shown that the prevalence of Mets in patients with moderate to severe OSA is 17.2%, a 2.5-fold increase in risk compared to control groups (18). Intermittent hypoxia (IH) is the primary cause of metabolic dysfunction in these patients. IH may induce metabolic disorders by impairing pancreatic *β*-cell function, causing inflammatory responses in adipose tissue, and increasing glucose production in the liver, which interferes with insulin signaling pathways and disrupts glucose metabolism (19, 20). As a result, patients with overlap syndrome may be more susceptible to metabolic disorders. The findings of this study revealed significantly higher levels of erythrocytes, hemoglobin, platelets, platelet distribution width, total cholesterol, and fasting blood glucose in overlap syndrome patients. Additionally, the prevalence of Mets in overlap syndrome patients was 55.24%, suggesting that overlap syndrome may be more prone to metabolic disorders and is closely associated with the development of conditions such as hyperglycemia. However, larger-scale studies are needed.

To clarify the risk factors associated with the development of metabolic syndrome (Mets) in patients with OVS, this study categorized participants into two groups: those with Mets and those without. The results showed that OVS patients with Mets exhibited significantly higher levels of inflammation markers, along with more severe hypoxic state than OVS without Mets patients. This is consistent with that OSA patients with more severe intermittent hypoxia are more prone to developing metabolic syndrome, while patients with Mets exhibit higher levels of inflammation markers (21, 22). As a key pathological feature, the core components of Mets—including obesity, insulin resistance, and dyslipidemia—collectively trigger a state of chronic low-grade inflammation in the body. For example, adipocytes, particularly visceral adipocytes, secrete large amounts of proinflammatory cytokines such as tumor necrosis factor-*α* and interleukin-6. These cytokines stimulate hepatic synthesis of CRP, thereby contributing to elevated circulating levels of this inflammatory marker (23). Studies showed that an elevated CRP level serves as a key marker indicating an increased risk of cardiovascular disease in patients with metabolic syndrome (24).

Our results also revealed that the mean SpO_2_, baseline SpO_2_, SpO_2_ below 90%, and SpO_2_ below 80% may be risk factors for Mets in OVS patients, indicating that hypoxia may play a role in the development of Mets in these patients. However, inflammatory indicators such as Neutrophil cell, RDW, MHR, NHR, NLR, and CRP were not identified as risk factors for OVS with Mets. Our findings suggest that inflammation may not play a significant role in the development of Mets associated with OVS, or it may only be a minor factor. Notably, the observed lack of statistical significance for inflammatory markers in our analysis could partially reflect limitations in our study’s sample size. A modest sample size may reduce statistical power, potentially masking subtle but biologically relevant associations between specific inflammatory pathways and Mets risk in OVS patients. This is particularly relevant given that some inflammatory indices did show numerical elevations, even if they did not reach statistical significance—hinting at a potential signal that requires further validation. Recognizing this uncertainty, larger cohorts needed for more robust subgroup analyses (e.g., stratifying by OVS severity or altitude) and may help clarify whether inflammation exerts context-dependent effects that were not detectable in our current dataset. Additionally, future studies could incorporate more comprehensive assessments of inflammatory mediators (e.g., cytokines, acute-phase proteins) and longitudinal designs to better characterize the temporal relationship between inflammation and Mets onset in OVS.

It has been reported that individuals with pre-existing asymptomatic obstructive sleep apnea (OSA) are more likely to experience reduced SpO2 and clinical deterioration (25). Additionally, hypobaric hypoxia at high altitudes impairs systemic tissue oxygenation, which in turn induces insulin resistance and a metabolic shift-marked by decreased oxidative phosphorylation and glucose storage, together with enhanced glycolysis. Notably, the induction of insulin resistance is not exclusive to hypobaric hypoxia; it also occurs in healthy individuals exposed to normobaric hypoxia and in patients with obstructive sleep apnea (26). Intermittent hypoxia, one of the primary pathologies of OSA, subjects cells to repeated cycles of hypoxia and normoxia, leading to oxidative stress and systemic inflammation. This, in turn, may contribute to the development of type 2 diabetes/insulin resistance, obesity, hypertension, and dyslipidemia in Mets through the regulation of gene expression and oxidative stress (20, 27). Furthermore, hypoxia can also lead to metabolic dysfunction (28–30). Studies have shown that hypoxia-induced transcription factors can contribute to the development of non-alcoholic fatty liver disease by regulating lipid metabolism in hepatocytes (30), and impaired carbohydrate and lipid metabolism (31). Therefore, the low-pressure and hypoxic environment at middle and high altitudes may further promote the development of Mets in OVS patients by reducing their blood SpO_2_ levels. The results of this study showed a higher prevalence of Mets in patients living above 2,500 meters compared to those living below 2,500 meters.

In conclusion, the prevalence of Mets in OVS patients in middle and high altitude areas was 55.24%, with a higher incidence of Mets in OVS patients living above 2,500 meters compared to those living below that altitude. Additionally, mean SpO_2_, baseline SpO_2_, and SpO_2_ levels below 90 and 80% may be risk factors for Mets in overlap syndrome patients.

## 5 Limitations

A major and unavoidable limitation of this study lies in its retrospective design, which inherently introduces several critical biases that significantly constrain the interpretation of our results. First, information bias is intrinsic to this approach: the data were extracted from clinical records compiled for patient care rather than research purposes, leading to inconsistencies in the documentation of variables such as symptom onset, treatment adherence, sociodemographic characteristics, and the distribution of laboratory test results. Second, selection bias represents a key concern: our sample was derived from patients who sought care at our institution, potentially overrepresenting those with more severe disease while excluding individuals with milder symptoms or undiagnosed cases. This limits the generalizability of our findings to broader populations. Most importantly, retrospective designs cannot establish causality. While we observed an association between high altitude and metabolic syndrome, unmeasured confounding factors—such as dietary habits, physical activity levels, and other unhealthy lifestyle behaviors not documented in the records—may account for this relationship. This represents a key limitation that prospective, controlled studies are uniquely positioned to resolve. These constraints underscore the necessity of future prospective investigations to validate our findings.

## Data Availability

The original contributions presented in the study are included in the article. Further inquiries can be directed to the corresponding author.
