# Correction: Clinical characteristics and risk factor analysis of overlapping syndromes of COPD-OSA with metabolic syndrome in middle-to-high altitude areas

**DOI:** 10.3389/fmed.2025.1737539

**Published:** 2025-12-17

**Authors:** Yisha Qin, Aniu Aju, Yilin Wan, Mengchen Song, Xingxing Hao, Jiwu Li, Xuefeng Shi

**Affiliations:** 1 Department of Clinical Medicine, Qinghai University, Xining, China; 2 Respiratory and Critical Care Medicine Department, Luoyang First People's Hospital, Luoyang, China; 3 Respiratory and Critical Care Medicine Department, Qinghai Provincial People's Hospital, Xining, China

**Keywords:** middle-high altitude, overlapping syndromes, metabolic syndrome, risk factors, clinical characteristics

This article was erroneously submitted as: Review article type. The correct article type is Original Research.

The **Ethics statement** has been added accordingly and appears below:

“The studies involving humans were approved by the ethics committee Qinghai provincial people's hospital (NO. 2024-024-02). The studies were conducted in accordance with the local legislation and institutional requirements. Written informed consent to participate in this study was not required from the participants in accordance with the national legislation and the institutional requirements and due to the retrospective nature of the study.”

The **Data Availability statement** has been added accordingly and appears below:

“The original contributions presented in the study are included in the article. Further inquiries can be directed to the corresponding author.”

The original version of this article has been updated.

